# Author Correction: Identification of recurrent *USP48* and *BRAF* mutations in Cushing’s disease

**DOI:** 10.1038/s41467-023-40833-6

**Published:** 2023-08-23

**Authors:** Jianhua Chen, Xuemin Jian, Siyu Deng, Zengyi Ma, Xuefei Shou, Yue Shen, Qilin Zhang, Zhijian Song, Zhiqiang Li, Hong Peng, Cheng Peng, Min Chen, Cheng Luo, Dan Zhao, Zhao Ye, Ming Shen, Yichao Zhang, Juan Zhou, Aamir Fahira, Yongfei Wang, Shiqi Li, Zhaoyun Zhang, Hongying Ye, Yiming Li, Jiawei Shen, Hong Chen, Feng Tang, Zhenwei Yao, Zhifeng Shi, Chunjui Chen, Lu Xie, Ye Wang, Chaowei Fu, Ying Mao, Liangfu Zhou, Daming Gao, Hai Yan, Yao Zhao, Chuanxin Huang, Yongyong Shi

**Affiliations:** 1 Shanghai Key Laboratory of Psychotic Disorders, Shanghai Mental Health Center, Shanghai Jiao Tong University School of Medicine; Bio-X Institutes, Key Laboratory for the Genetics of Developmental and Neuropsychiatric Disorders (Ministry of Education), and the Collaborative Innovation Center for Brain Science, Shanghai Jiao Tong University, Shanghai, 200030 China; 2 Department of Otolaryngology Head and Neck Surgery & Center of Sleep Medicine, Shanghai Jiao Tong University Affiliated Sixth People’s Hospital, Shanghai, 200233 China; 3 Shanghai Institute of Immunology, Key Laboratory of Cell Differentiation and Apoptosis of Chinese Ministry of Education, Shanghai Jiao Tong University School of Medicine, Shanghai, 200025 China; 4 Department of Neurosurgery, Huashan Hospital, Shanghai Medical College, Fudan University, Shanghai, 200040 China; 5 Shanghai Pituitary Tumor Center, Shanghai, 200040 China; 6 Institute of Neurosurgery, Fudan University, Shanghai, 200040 China; 7 CAS Key Laboratory of Systems Biology, CAS Center for Excellence in Molecular Cell Science, Innovation Center for Cell Signaling Network, Shanghai Institute of Biochemistry and Cell Biology, Chinese Academy of Sciences, Shanghai, 200031 China; 8 University of Chinese Academy of Sciences, Beijing, 100049 China; 9 Drug Discovery and Design Center, State Key Laboratory of Drug Research, Shanghai Institute of Materia Medica, Chinese Academy of Sciences, Shanghai, 201203 China; 10 Department of Endocrinology, Huashan Hospital, Shanghai Medical College, Fudan University, Shanghai, 200040 China; 11 Department of Pathology, Huashan Hospital, Shanghai Medical College, Fudan University, Shanghai, 200040 China; 12 Department of Radiology, Huashan Hospital, Shanghai Medical College, Fudan University, Shanghai, 200040 China; 13 Shanghai Center for Bioinformation Technology (SCBIT), Shanghai Academy of Science and Technology, Shanghai, 201203 China; 14 Department of Epidemiology, School of Public Health, Fudan University, Shanghai, 200032 China; 15 State Key Laboratory of Medical Neurobiology, Institute of Neurosurgery, Shanghai Medical College, Fudan University, 200040 Shanghai, China; 16 Department of Pathology, Preston Robert Tisch Brain Tumor Center, Duke University Medical Center, Durham, NC 27710 USA; 17 Institute of Neuropsychiatric Science and Systems Biological Medicine, Shanghai Jiao Tong University, Shanghai, 200030 China; 18 Department of Psychiatry, First Teaching Hospital of Xinjiang Medical University, Urumqi, Xinjiang 830054 China; 19 The Affiliated Hospital of Qingdao University & The Biomedical Sciences Institute of Qingdao University (Qingdao Branch of SJTU Bio-X Institutes), Qingdao University, Qingdao, Shandong 266003 China

Correction to: *Nature Communications* 10.1038/s41467-018-05275-5, published online 09 August 2018

The original article contained an error made during the assembly of Fig. 2b. In Fig. 2b, the same BRAF IHC picture from the BRAF mutated sample was selected by mistake for the two panels shown.

The corrected version of Fig. 2b is shown below.
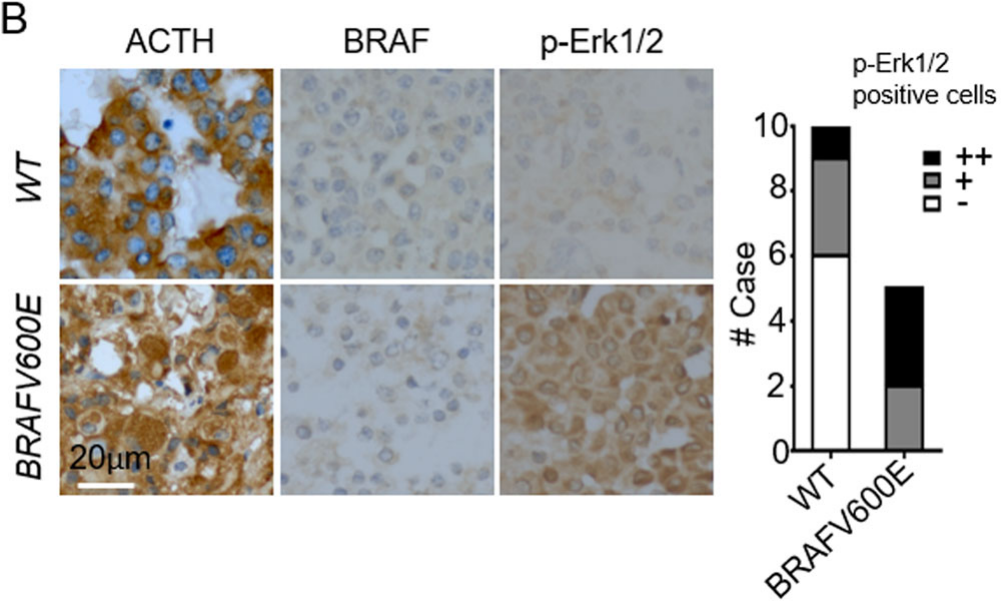


The figure has been corrected in the PDF and HTML versions of the article.

A pdf file containing the raw data for the same panels is appended below.

### Supplementary information

Supplementary Raw Data

