# Supplementary material for: Identification of recurrent USP48 and BRAF mutations in Cushing’s disease
Source: Nat Commun. 2018 Aug 9;9:3171. doi: 10.1038/s41467-018-05275-5 (PMC6085354; doi:10.1038/s41467-018-05275-5)
Supplement: Supplementary file 1 — Supplementary Information [file 41467_2018_5275_MOESM1_ESM.pdf]

Identification of recurrent *USP48* and *BRAF* mutations in Cushing's disease

Chen et al.

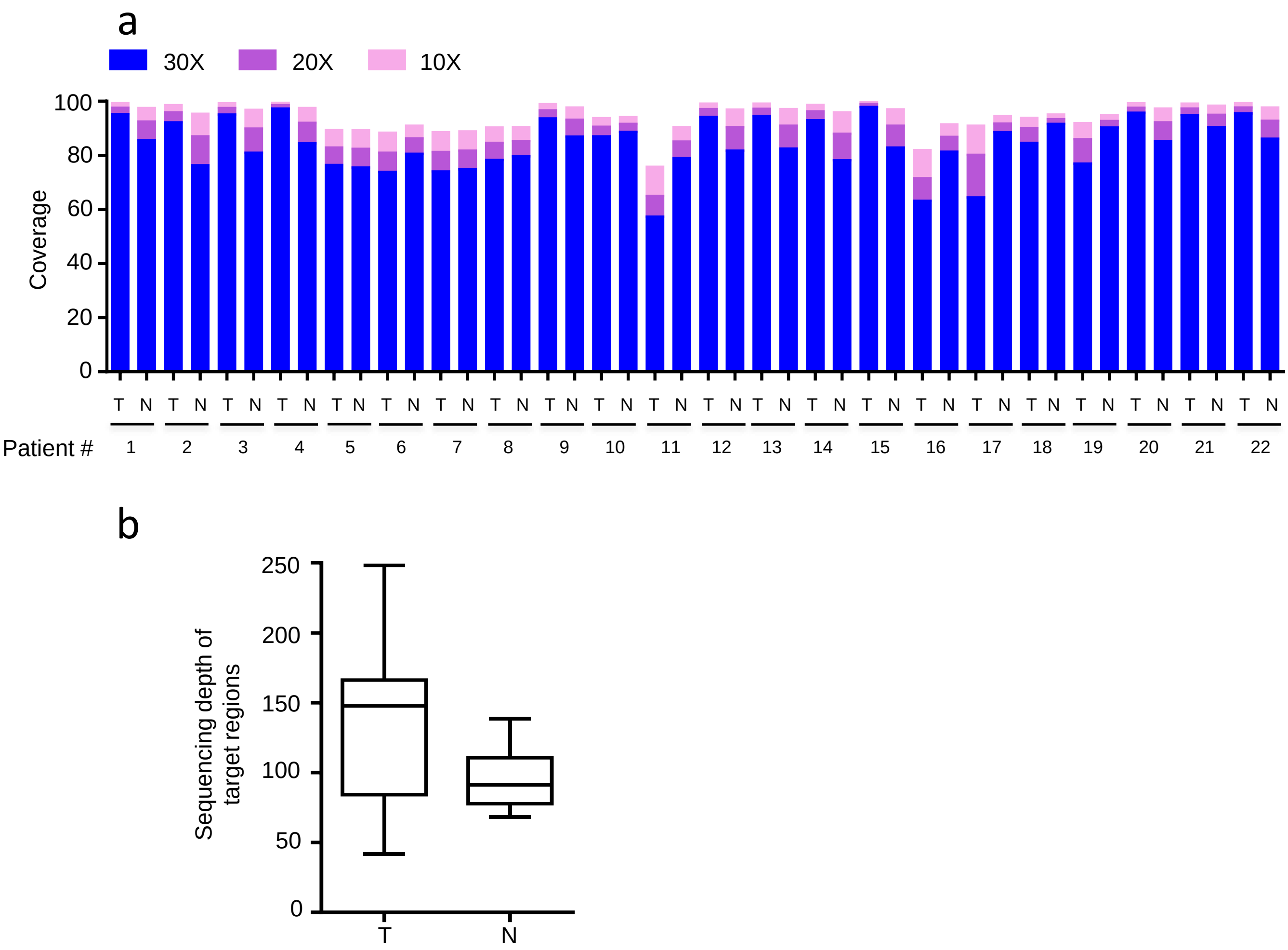

Supplementary Figure 1. Depth and coverage of whole-exome sequencing of target regions of paired tumor (T) and normal (N) DNA from 22 patients with Cushing's disease. Panel a shows the fractions of target bases covered by 30X reads, 20X reads or 10X reads each sample. Panel b box plots show the distribution of fold coverages of target regions. The centre lines in boxes represent the medians, the boxes limits represent the lower and upper quartile limits, and the whiskers represent the max and min fold coverage.

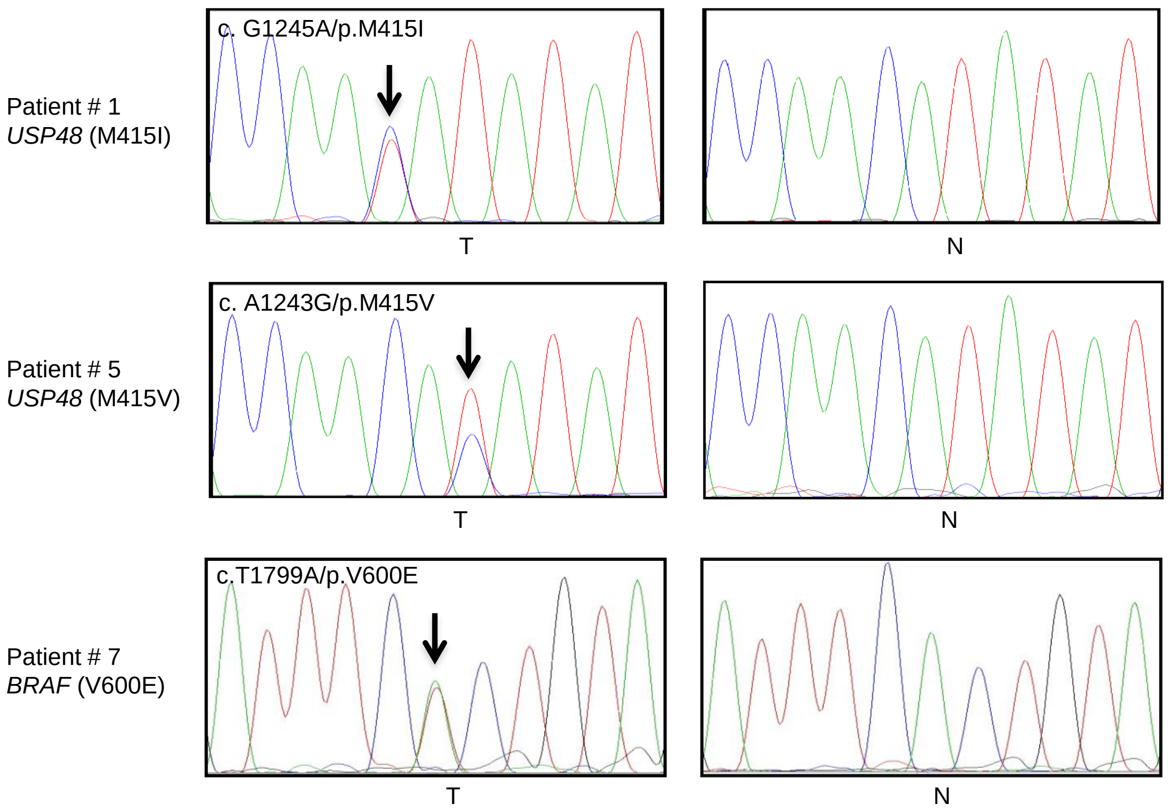

Supplementary Figure 2. Validation of *USP48* and *BRAF* mutations detected by whole-exome sequencing. The data is presented from Sanger sequencing. T, DNA from tumor; N, DNA from paired normal blood. Mutated bases are indicated by arrows.

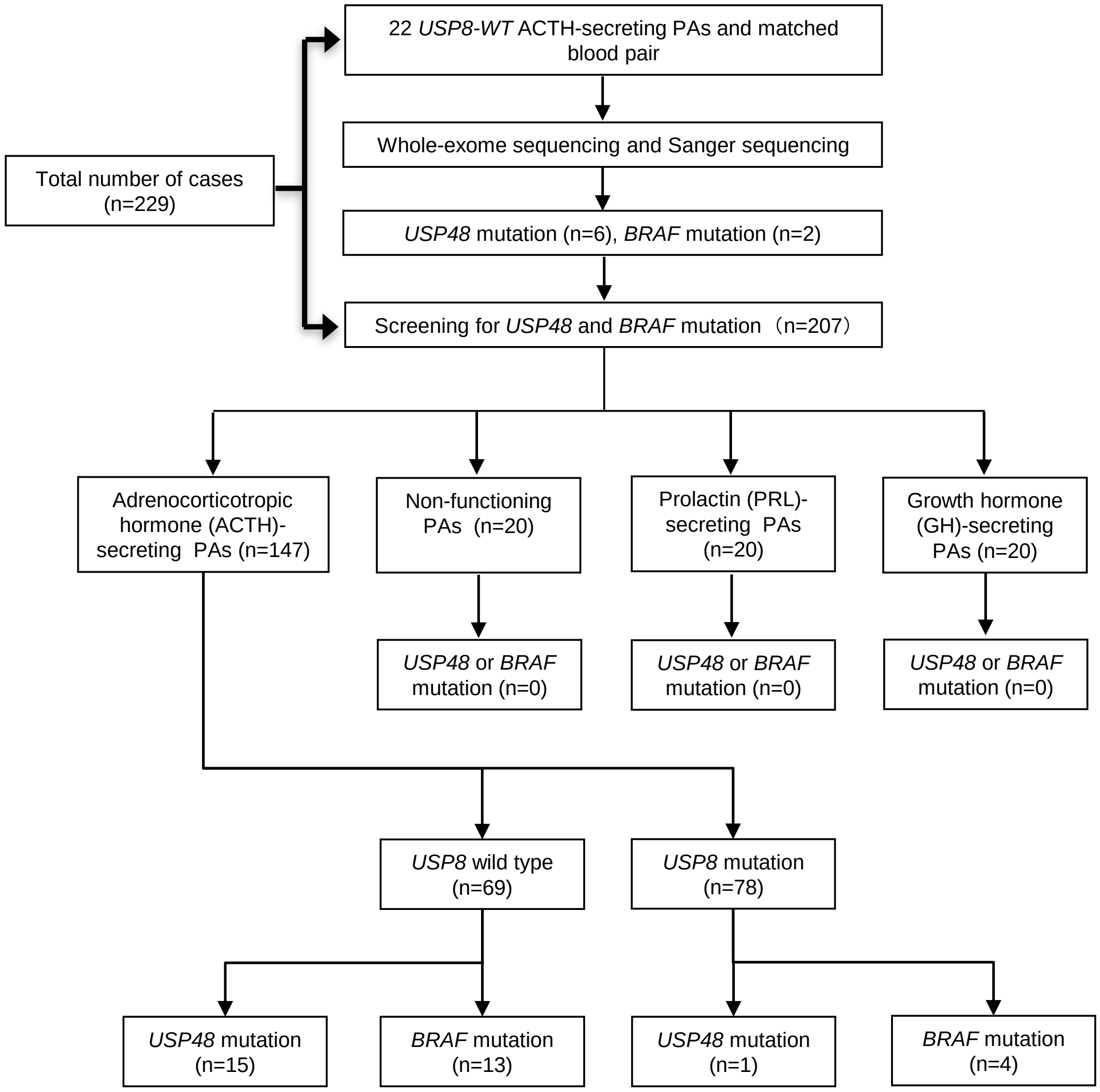

Supplementary Figure 3. Overview of the patients included in this genetic study and summary of *USP8* mutational status of these patients. Data are based on whole-exome sequencing and Sanger sequencing validation. The number of patients is indicated by n.

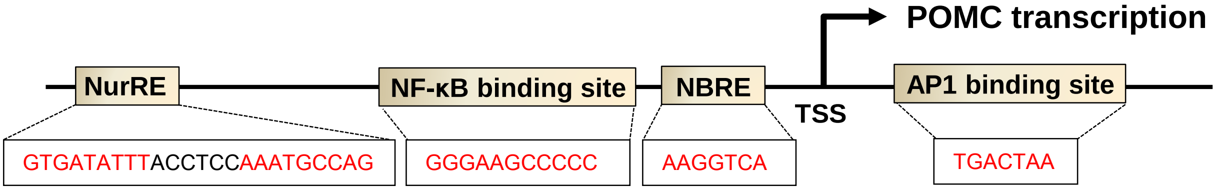

Supplementary Figure 4. Schematic diagram showing the partial of the *POMC* gene promoter. The location and sequence of the NurRE, NF-κB, NBRE and AP1 binding sites in the *POMC* promoter are depicted. TSS, transcription start site.

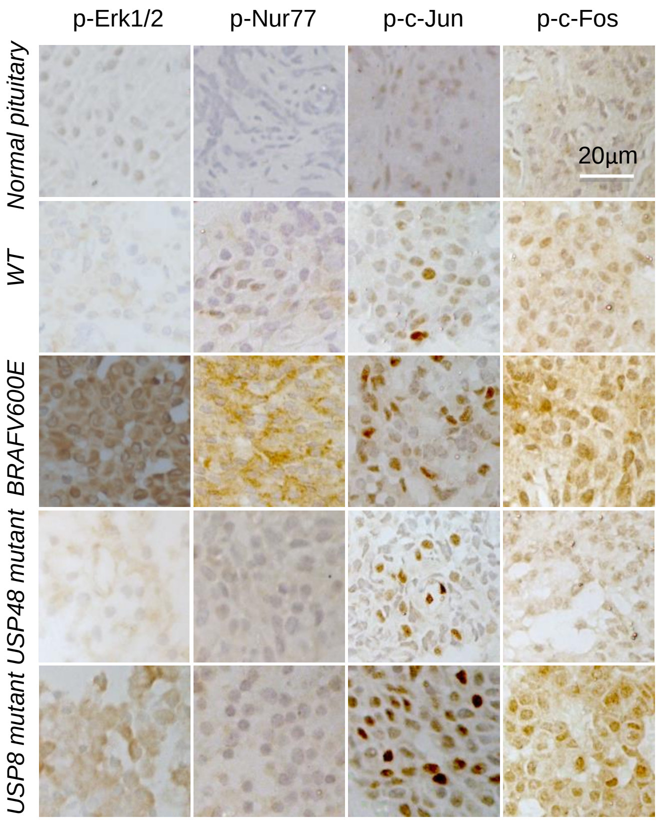

Supplementary Figure 5. Immunohistochemcial staining of indicated proteins in human normal pituitary and corticotroph adenomas with indicated genotypes.

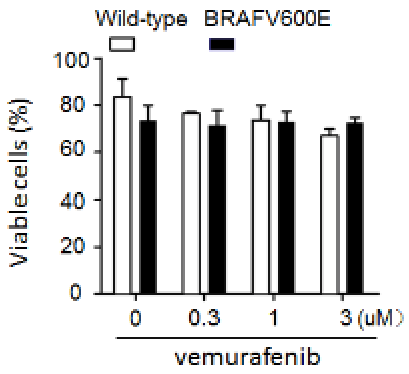

Supplementary Figure 6. Human corticotroph adenoma primary cell cultures carrying wild-type or mutated *BRAF* were treated with vemurafenib at different doses for 48 h. The viable cells were determined by exclusion of PI. Error bars represent SEM of three measurements.

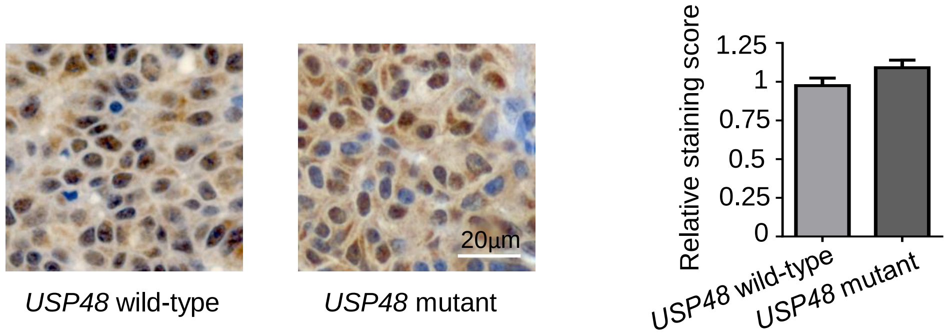

Supplementary Figure 7. Immunohistochemcial staining of USP48 in human corticotroph adenomas with three wild-type and three *USP48* mutated samples. Representative images are shown on the left and statistical data are shown on the right. Error bars represent standard deviation between three measurements.

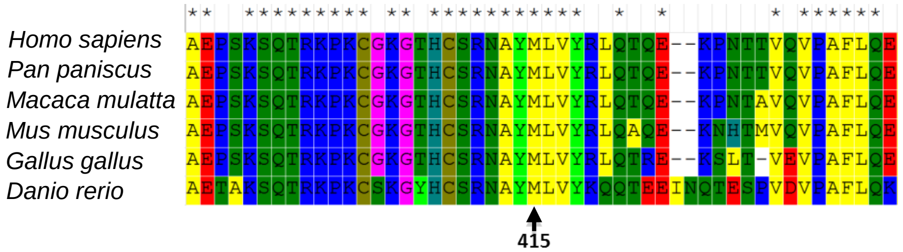

Supplementary Figure 8. Sequence alignment of partial USP48 protein across distinct species. M415 is indicated.

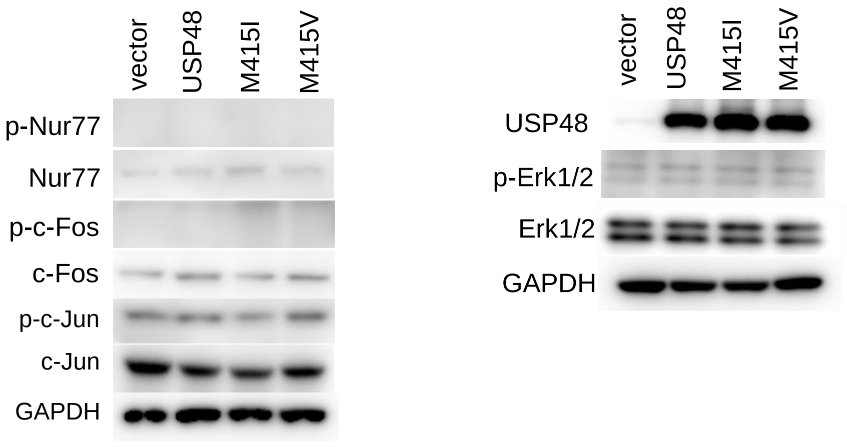

Supplementary figure 9. Western blot analysis of indicated proteins in AtT-20 cells stably transfected with indicated expression vectors.

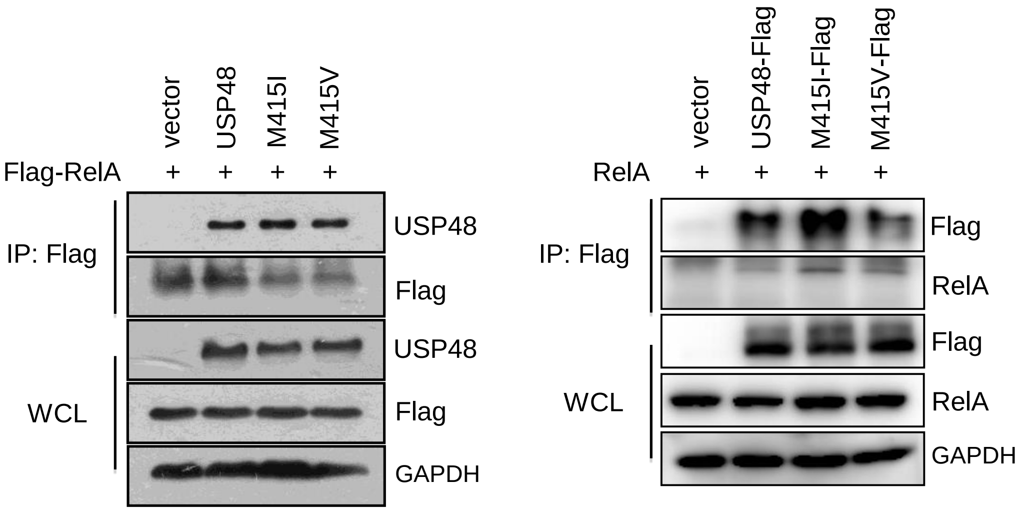

Supplementary Figure 10. RelA interacts with wild-type and mutated *USP48*. HEK293T cells were co-transfected with RelA and wild-type or mutated *USP48*, immunoprecipitated with Flag beads and immunoblotted with indicated antibodies.

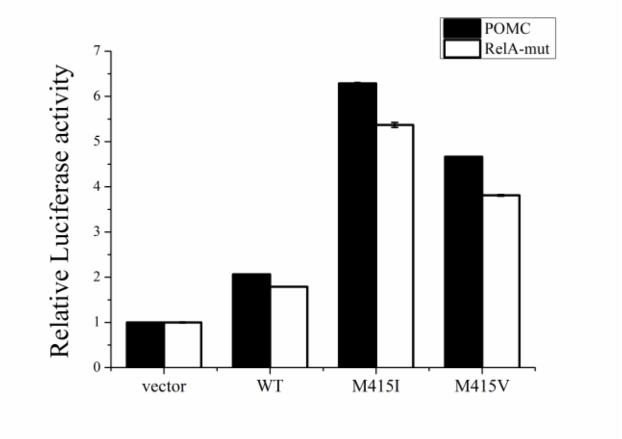

Supplementary Figure 11. Activities of *POMC* or mutated *RelA* promoters in AtT-20 cells transfected with wild-type or *USP48* M415I/V together with a control vector expressing renilla luciferase. Data are ratios of luciferase activity/renilla activity. Error bars represent standard deviation between three measurements.

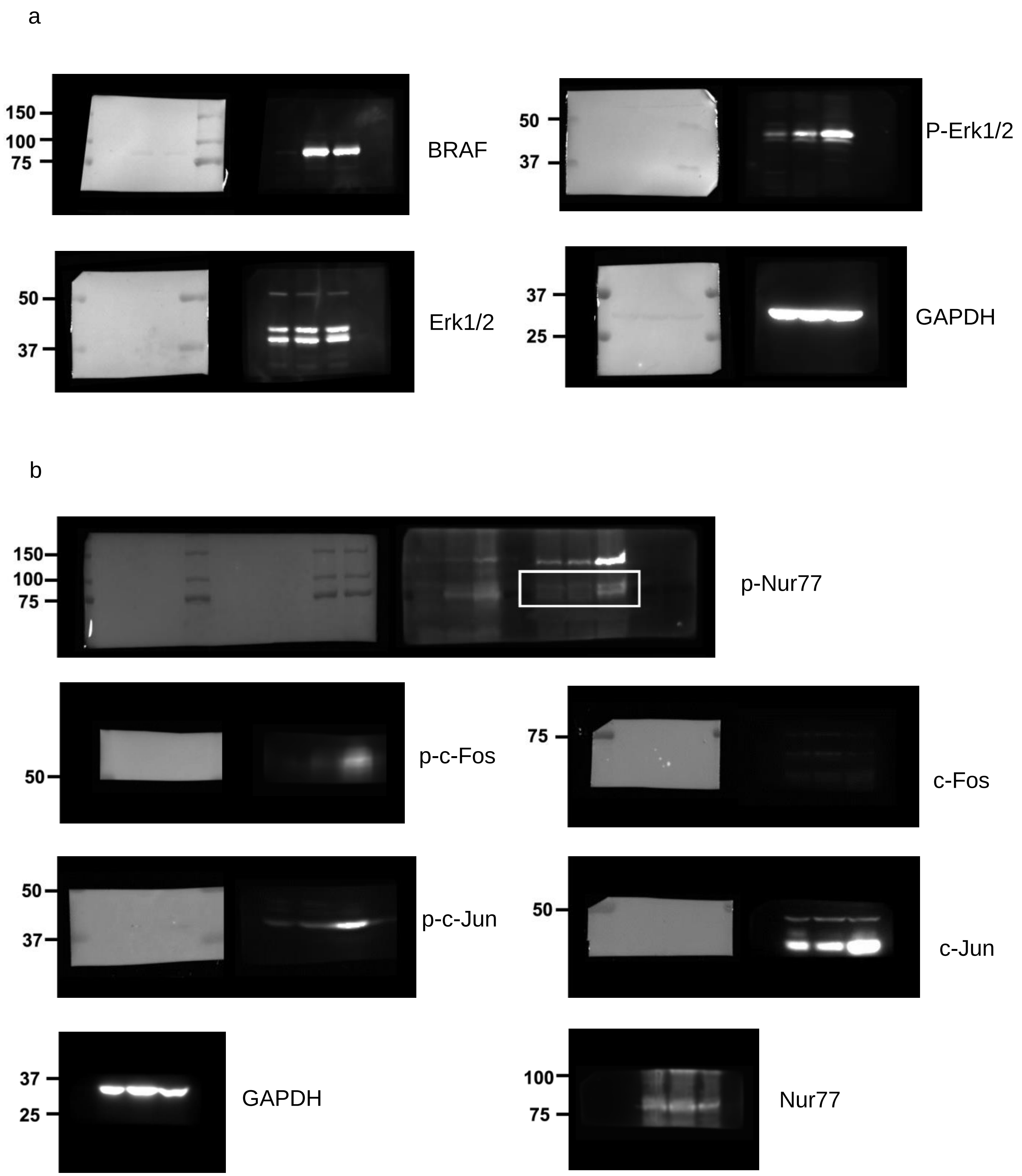

Supplementary Figure 12. Uncropped images of the blots.

a, Western blots shown in Figure 2a

b, Western blots shown in Figure 2c

Supplementary Table 1. The list of genes mutated in at least two cases out of 22 corticotrophin adenomas.

mutated gene	mutated cases
USP48	6
BRAF	2
NR3C1	2
HCFC1	2

Supplementary Table 2. The details of *USP8* mutations identified in 169 corticotrophin adenomas.

Position	Allele	Gene	Transcript	cDNA_pos	cds_pos	aa_pos	AA_change	Detail	n
chr1:22056252	C/A	USP48	NM_032236	1483	1245	415	M>I	nonsyn	2(1.18%)
chr1:22056252	C/T	USP48	NM_032236	1483	1245	415	M>I	nonsyn	10(5.92%)
chr1:22056254	T/C	USP48	NM_032236	1481	1243	415	M>V	nonsyn	4(2.37%)
chr1:22056257	A/G	USP48	NM_032236	1478	1240	414	Y>H	nonsyn	1(0.59%)
chr1:22056265	C/T	USP48	NM_032236	1470	1232	411	R>Q	nonsyn	1(0.59%)
chr1:22056281	C/T	USP48	NM_032236	1454	1216	406	G>R	nonsyn	1(0.59%)
chr1:22056289	C/T	USP48	NM_032236	1446	1208	403	C>Y	nonsyn	1(0.59%)
chr1:22056290	A/T	USP48	NM_032236	1445	1207	403	C>S	nonsyn	1(0.59%)
chr1:22056322	T/C	USP48	NM_032236	1413	1175	392	E>G	nonsyn	1(0.59%)

Supplementary Table 3.

Primers for reporters

POMC-F: 5- GGCGCTAGCGCCATGATTCTTGACAGCCTCT -3
POMC-R: 5- GAGCTCGAGACACCCTTACCTGTCGCGGAA -3
POMC-NUR2-F: 5- ACCCCCTCCTCATTATCCTACGAGACCTCCCAGCTAGTCGAAGGCAGATGGACGCACACAGGTAATTC -3
POMC-NUR2-R: 5- TCCATCTGCCTTCGACTAGCTGGGAGGTCTCGTAGGATAATGAGGAGGGGGTCTGTTACGCA -3
POMC-RELA-MUT-F: 5- CAGCCCCGACCGTTAACGCAACCCTCCCGCGGCCCGCCGCCCC -3
POMC-RELA-MUT-R: 5- CCGCGGGAGGGTTGCGTTAACGGTCGGGGCTGCGCGCACAGCA -3

Primers for constructs

BRAF-F: 5- GCTGCGGCCGCGTGGACAGGAAACGCACCATA -3
BRAF-R: 5- GGCGCTAGCGCCACCATGGCGGCGCTGAGCGGTGGCG -3
USP48-F: 5- GCCGAATTCGCCACCATGGCCCCGCGGCTGCAGCTG -3
USP48-R: 5- GTGGCGGCCGCTTA ATGTCCAAGAAGACCAGTACC -3
M415V-F: 5- CGAAATGCATATGTGTTGGTTTATAGACTGCAAACT -3
M415V-R: 5- TATAAACCAACACATATGCATTTCGAGAGCAATG -3
M415I-F: 5- CGAAATGCATATATCTTGGTTTATAGACTGCAAACT -3
M415I-R: 5- TATAAACCAAGATATATGCATTTCGAGAGCAATG -3

Primers for identification of the mutation status of *BRAF* and *USP48*

BRAF-F: 5- GGCCAAAAATTTAATCAGTGGA -3
BRAF-R: 5- CATAATGCTTGCTCTGATAGGA -3
USP48-F: 5- TGCCTGCTATAATCCTGGAAA -3
USP48-R: 5- TCAGCAGAACCTTCTAAGTCTCA -3
