# Supplementary material for: Identification of recurrent USP48 and BRAF mutations in Cushing’s disease
Source: Nat Commun. 2018 Aug 9;9:3171. doi: 10.1038/s41467-018-05275-5 (PMC6085354; doi:10.1038/s41467-018-05275-5)
Supplement: Supplementary file 3 — Description of Additional Supplementary Files [file 41467_2018_5275_MOESM3_ESM.pdf]

Description of Additional Supplementary Files

Supplementary Data 1. Overview of mutations found in individual tumor sample upon exome sequencing are illustrated.
