# Supplementary material for: Author Correction: Identification of recurrent USP48 and BRAF mutations in Cushing’s disease
Source: Nat Commun. 2023 Aug 23;14:5128. doi: 10.1038/s41467-023-40833-6 (PMC10447516; doi:10.1038/s41467-023-40833-6)
Supplement: Supplementary file 1 — Supplementary Raw Data [file 41467_2023_40833_MOESM1_ESM.pdf]

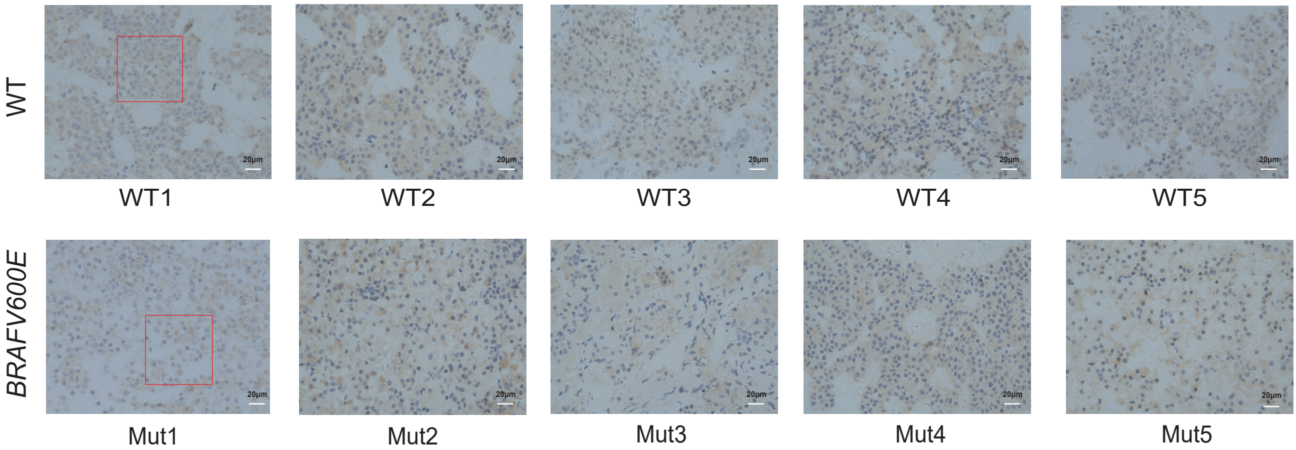

Figure 1. Immunohistochemistry of BRAF protein expression in five wild-type (WT1-5) and five *BRAF V600E* (Mut1-5) tumors
